# Plagues Upon the Earth: Disease and the Course of Human History

**DOI:** 10.3201/eid2809.220812

**Published:** 2022-09

**Authors:** W. Clyde Partin

**Affiliations:** Emory University, Atlanta, Georgia, USA; Editorial Board member, Emerging Infectious Diseases, Atlanta

**Keywords:** bacteria, viruses, zoonoses, plague, Kyle Harper, John Donne, kissing bug, genomics, evolution

Daniel Defoe’s 1722 novel, A Journal of the Plague Year (Figure)*,* which chronicles London’s 1664–65 bubonic plague, is a superb starting point for books about plagues. Since then, >2,000 books about plagues have been penned. Harper’s “claim to novelty rests in part on the effort to draw from a new source of knowledge: genomes,” which he boldly states in his 15-page introduction. This goal is marvelously achieved in the next 494 pages. Another 160 pages of notes and references accentuate the detailed nature of this tome. 

**Figure:**
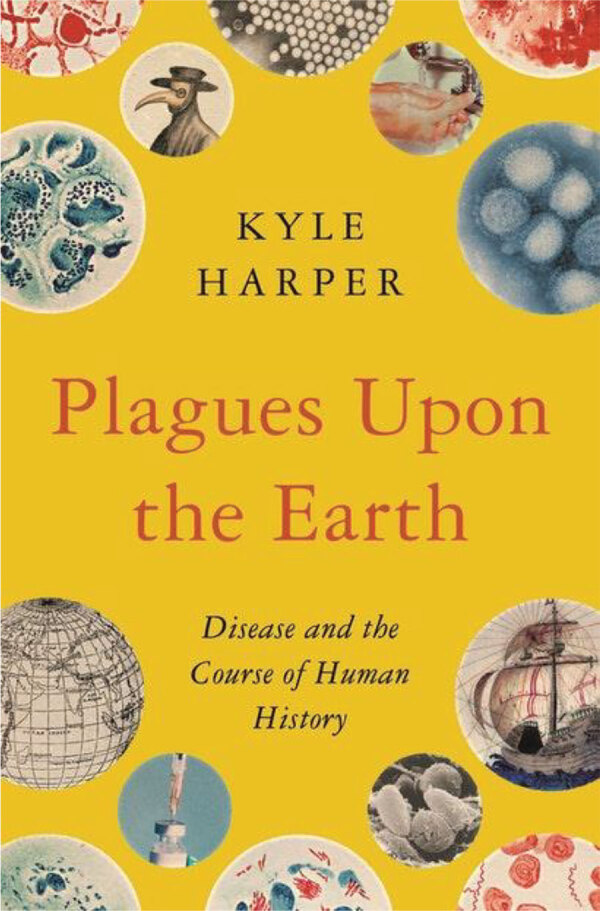
Plagues Upon the Earth: Disease and the Course of Human History

The work is anchored around 4 transformative energy revolutions: mastery of fire, invention of farming, regular transatlantic crossings, and harnessing fossil fuels. The variations of disease burden and the imprint of endemics and epidemics on human history become the compelling story. The author quotes biologist E.O. Wilson, who favored consilience: harnessing knowledge from multiple domains to arrive at a unified explanation. Plagues Upon the Earth comes as close as possible to consilience to explain the cruel march against humanity engendered by infectious diseases.

The first section, Fire, discusses evolution and genetics in the microbiology world. Mastery of fire “allowed our ancestors to disperse out of Africa and settle from the equator to the Arctic.” Creative use of fire promoted human adaptation to various climates and infectious agents contaminating the environment and food sources. The second section, Farms, is informative, especially the chapter Dung and Death. The author’s portrayal of the lowly house fly might haunt your next summer picnic. This segment includes Darwin’s remarkable description of the kissing bug. Section 3, Frontiers, unpacks a plethora of plague information. The chapter Of Lice and Men artfully depicts John Donne’s Devotions upon Emergent Occasions, which Harper considers “one of the most beautiful meditations on sickness ever written,” and includes the literary gems “no man is an island” and “never send to know for whom the bell tolls; it tolls for thee.”

The amalgamation of humanities and science is refreshing. The last section, Fossils, melds economics, global health, disease, and technology. Harper marvels over what economist Angus Deaton calls the “Great Escape,” the ability of humankind to rise above the “doom of poverty and early death.” This ability and the enhanced technical capacity to prevent, diagnose, and treat disease are associated with life expectancy elongation. However, Harper’s lucid counterstatement is sobering. He states, “The evolution of pathogens is the basic reason we can never entirely escape the risk of global pandemics.”

The writing is clear and straightforward in an organized, breezy, digestible style. Scientific and genetic concepts are logically presented. Well-placed metaphors, analogies, and quotations are used effectively to augment the point being made. For example, a 1921 appraisal of mosquitoes by English physician and writer Havelock Ellis asks us, “If you would see all of Nature gathered up at one point, in all her loveliness, and her skill, and her deadliness, and her sex, where would you find a more exquisite symbol than the mosquito?” 

Anyone with an interest in science history, microbial genetics, evolution, and understanding plagues will find this a worthy and enlightening read. Molecular biologists and evolutionary geneticists might find certain explanations somewhat simplified, but always well crafted.

